# Toll-Like Receptor 7 Agonist GS-9620 Induces HIV Expression and HIV-Specific Immunity in Cells from HIV-Infected Individuals on Suppressive Antiretroviral Therapy

**DOI:** 10.1128/JVI.02166-16

**Published:** 2017-03-29

**Authors:** Angela Tsai, Alivelu Irrinki, Jasmine Kaur, Tomas Cihlar, George Kukolj, Derek D. Sloan, Jeffrey P. Murry

**Affiliations:** Gilead Sciences, Foster City, California, USA; Emory University

**Keywords:** latency, TLR7, broadly neutralizing antibodies, cytotoxic T lymphocytes, human immunodeficiency virus, interferon alpha, interferons, plasmacytoid dendritic cells, reservoir

## Abstract

Antiretroviral therapy can suppress HIV replication to undetectable levels but does not eliminate latent HIV, thus necessitating lifelong therapy. Recent efforts to target this persistent reservoir have focused on inducing the expression of latent HIV so that infected cells may be recognized and eliminated by the immune system. Toll-like receptor (TLR) activation stimulates antiviral immunity and has been shown to induce HIV from latently infected cells. Activation of TLR7 leads to the production of several stimulatory cytokines, including type I interferons (IFNs). In this study, we show that the selective TLR7 agonist GS-9620 induced HIV in peripheral blood mononuclear cells (PBMCs) from HIV-infected individuals on suppressive antiretroviral therapy. GS-9620 increased extracellular HIV RNA 1.5- to 2-fold through a mechanism that required type I IFN signaling. GS-9620 also activated HIV-specific T cells and enhanced antibody-mediated clearance of HIV-infected cells. Activation by GS-9620 in combination with HIV peptide stimulation increased CD8 T cell degranulation, production of intracellular cytokines, and cytolytic activity. T cell activation was again dependent on type I IFNs produced by plasmacytoid dendritic cells. GS-9620 induced phagocytic cell maturation and improved effector-mediated killing of HIV-infected CD4 T cells by the HIV envelope-specific broadly neutralizing antibody PGT121. Collectively, these data show that GS-9620 can activate HIV production and improve the effector functions that target latently infected cells. GS-9620 may effectively complement orthogonal therapies designed to stimulate antiviral immunity, such as therapeutic vaccines or broadly neutralizing antibodies. Clinical studies are under way to determine if GS-9620 can target HIV reservoirs.

**IMPORTANCE** Though antiretroviral therapies effectively suppress viral replication, they do not eliminate integrated proviral DNA. This stable intermediate of viral infection is persistently maintained in reservoirs of latently infected cells. Consequently, lifelong therapy is required to maintain viral suppression. Ultimately, new therapies that specifically target and eliminate the latent HIV reservoir are needed. Toll-like receptor agonists are potent enhancers of innate antiviral immunity that can also improve the adaptive immune response. Here, we show that a highly selective TLR7 agonist, GS-9620, activated HIV from peripheral blood mononuclear cells isolated from HIV-infected individuals with suppressed infection. GS-9620 also improved immune effector functions that specifically targeted HIV-infected cells. Previously published studies on the compound in other chronic viral infections show that it can effectively induce immune activation at safe and tolerable clinical doses. Together, the results of these studies suggest that GS-9620 may be useful for treating HIV-infected individuals on suppressive antiretroviral therapy.

## INTRODUCTION

Human immunodeficiency virus (HIV) infection can be well controlled by current antiretroviral therapy (ART). However, people living with HIV require lifelong therapy because treatment interruption inevitably leads to viral recrudescence. The virus that emerges following treatment interruption is likely derived from latent reservoirs, such as long-lived memory CD4 T cells (1, 2). Integrated HIV proviral DNA in these infected cells may exist in at least two states: (i) a transcriptionally competent form with the potential to produce virus and (ii) a quiescent form that allows infected cells to evade the immune response but that may become activated at a later time (3, 4). The former state contributes to the low-level viremia and viral antigenemia that persist despite ART and to increased risk for long-term comorbidities, such as cardiovascular and renal disease (5–7). Therefore, there is a need to find therapeutic interventions that can induce long-lasting control or elimination of viral reservoirs.

A proposed strategy for eliminating viral reservoirs invokes a sequential “kick and kill” process. The method (administered during suppressive ART) requires an agent that can stimulate latent HIV expression so that the infected cells will in turn succumb to viral cytopathic effect or HIV-specific immune clearance. Clinical trials indicate that histone deacetylase (HDAC) inhibitors, such as vorinostat, panobinostat, and romidepsin, can induce cell-associated and/or plasma viral RNA (8–10). Many other agents, such as protein kinase C (PKC) agonists, interleukin 15 (IL-15) cytokine, and bromodomain inhibitors, have been tested using *in vitro* primary cell models. However, to date there is little evidence that stimulation of latent HIV expression, or “latency reversal,” can substantially reduce the latent viral reservoir (3, 11). This suggests that these approaches will need to be accompanied by a therapeutic intervention that facilitates immune-mediated clearance of infected cells (12, 13).

During the early course of most viral infections, antiviral immunity is induced through pattern recognition receptors, such as Toll-like receptors (TLRs), that stimulate the innate immune response. TLRs can trigger cytokine secretion, dendritic cell (DC) maturation, and antigen presentation, which in turn can enhance the adaptive immune response (14). In addition to boosting antiviral immunity, agonists of several TLRs, such as TLR1/2, TLR5, TLR8, and TLR9, have been shown to induce expression of latent HIV (15–18). Potentially, triggering this class of innate immune receptors may provide both the “kick” required to expose the latently infected cells and the immune responses required to “kill” them after latency reversal is induced.

TLR7 is predominantly found in the endosomal compartment of plasmacytoid dendritic cells (pDCs) and B cells (19–22). Agonists of the receptor have been identified and studied as vaccine adjuvants, antiviral agents, and antitumor therapeutics (23–26). Upon TLR7 stimulation, pDCs secrete copious amounts of type I interferons (IFNs), such as interferon alpha (IFN-α) and IFN-ω, that promote cell-autonomous antiviral defense through interferon-stimulated genes (ISGs). Type I IFNs also serve as a bridge between innate and adaptive immunity, enhancing antibody-dependent immunity and stimulating greater CD8^+^ T-cell responses (27, 28). GS-9620 is a potent TLR7-selective agonist that induces antiviral immunity and clearance of infection in preclinical models of hepatitis B virus infection (25, 26, 29). In clinical trials, oral administration of GS-9620 is safe and well tolerated at doses that stimulate ISG expression (30). Here, we demonstrate that GS-9620 induces HIV expression in cells from HIV-infected aviremic donors on ART through a mechanism that is dependent on type I IFNs. While the induction is modest compared to global T cell activators, they suggest that GS-9620 can be used to clinically test the hypothesis that extended dosing with safe yet moderately effective HIV RNA induction can meaningfully impact the HIV reservoir. We also show that GS-9620 enhances HIV-specific cellular cytotoxicity and anti-HIV antibody-mediated immunity to ultimately improve the killing of HIV-infected cells.

## RESULTS

### GS-9620 induces extracellular HIV RNA *ex vivo*.

**FIG 1:**
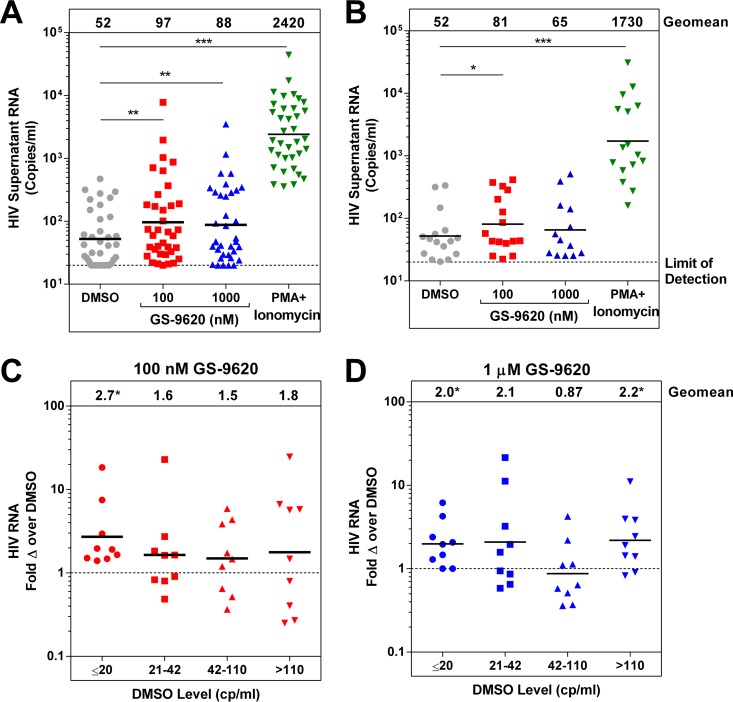
Stimulation of extracellular viral RNA from PBMCs treated with GS-9620. (A and B) Viral RNA was quantified in cell culture supernatants from PBMCs isolated from ART-suppressed HIV-infected individuals and treated with GS-9620 for 4 days. Each symbol represents the geometric mean for a single individual of 3 replicates (*n* = 41 individuals) (A) or 12 replicates (*n* = 16 individuals) (B). Significant differences from the vehicle (DMSO) control were determined by paired Student's *t* tests. (C and D) The same results shown in panel A were stratified based on the level of HIV RNA in the supernatant of the vehicle (DMSO) control condition. The data were normalized to DMSO values to calculate the fold change from the control in samples treated with 100 nM GS-9620 (C) or 1 μM GS-9620 (D). The horizontal bars indicate the geometric means, which are also shown at the top of each graph. Significant differences from 1 were determined by one-sample Student's *t* test. *, *P* < 0.05; **, *P* < 0.01; ***, *P* < 0.001.

To determine if GS-9620 is capable of inducing HIV RNA, we stimulated peripheral blood mononuclear cells (PBMCs) isolated from ART-suppressed HIV-infected individuals with the agonist and quantified HIV RNA in cell culture supernatants. We used supernatant HIV RNA as a surrogate for virion release from treated cells, as previously described (31). In the absence of stimulation, cells from 27 of 36 individuals produced detectable viral RNA (>20 copies/ml), despite the presence of antiretroviral drugs (Fig. 1; see Table S1 in the supplemental material). This is slightly lower than the rate of spontaneous virion production that we have seen in purified CD4 T cells. In a related study, CD4 T cells were isolated from 77 individuals with ART-suppressed HIV and cultured for 6 days at 5 million cells/ml. Spontaneous virion production was detectable in samples from 66 (86%) of the individuals (data not shown). This indicates that cells from most ART-suppressed HIV-infected individuals may spontaneously release low levels of HIV RNA, consistent with the residual viremia typically observed in ART-suppressed patients (32).

Treatment with GS-9620 increased HIV RNA production over a broad range of donors with a geometric mean of 1.9-fold at 100 nM (*P* = 0.0032 compared to paired vehicle-treated controls) and 1.7-fold at 1 μM GS-9620 (*P* = 0.0027 compared to paired vehicle-treated controls), using at least 3 replicates per condition in a cohort of PBMC samples from 36 donors (Fig. 1A; see Table S1 in the supplemental material). The median HIV RNA increase at 100 nM GS-9620 was 1.6-fold, with an interquartile range of 0.81- to 4.3-fold. The 100 nM concentration of GS-9620 increased HIV expression above the dimethyl sulfoxide (DMSO) control in cells from 69% of tested donors. These values likely represent an underestimation, as the measured supernatant HIV RNA from DMSO-treated control samples was undetectable in 9 donors (limit of detection, 20 copies/ml). Notably, among the 100 nM GS-9620-treated group, supernatant HIV RNA was undetectable in only 1 donor sample. The variation between replicates was substantial in these assays, with an average standard deviation across the triplicate vehicle controls of 0.26 log copies/ml. This is consistent with previous work showing that HIV RNA production from individual latently infected CD4 T cells can vary up to 100,000-fold (33). For this reason, we repeated the induction using 12 replicates in a second cohort of PBMC samples from 16 donors. Consistent with the first experiment, we observed a 1.6-fold increase in geometric mean viral RNA induction with 100 nM GS-9620 (*P* = 0.03 compared to vehicle-treated control) in the second, expanded replicate analysis (Fig. 1B; see Table S1 in the supplemental material).

The basal level of virion production could influence the effectiveness of latency reversal. For example, latency reversal may be most effective in cells that already have low-level HIV RNA expression. If so, the agent may be more active in individuals with high basal levels of HIV RNA. To determine if the basal level of supernatant HIV RNA affects latency reversal by GS-9620, we stratified the 36 individuals assessed in Fig. 1A into four groups based on the level of supernatant HIV RNA produced under the vehicle (DMSO) control conditions. For each group, we calculated the degree of change from the control condition that was found under the GS-9620-treated conditions. In each of the 4 groups at 100 nM GS-9620 and 3 of the 4 groups at 1 μM GS-9620, the geometric mean increased at least a 1.5-fold over the DMSO conditions (Fig. 1C and D). GS-9620 was at least as active in individuals with undetectable HIV RNA (≤20 copies/ml) under the DMSO conditions as in the other categories. There was no statistically significant difference in response to GS-9620 between the groups. This indicates that GS-9620 is unlikely to be preferentially active in individuals with high basal levels of virion production.

**FIG 2:**
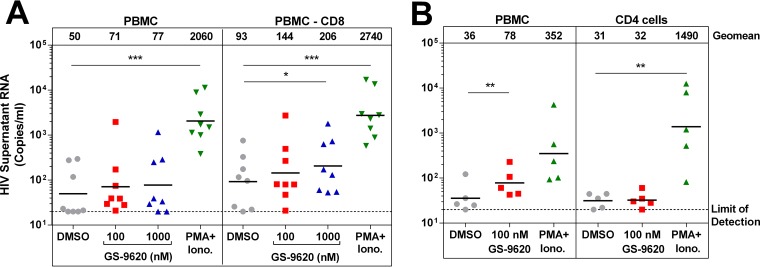
Treatment of PBMCs depleted of CD8 cells or isolated CD4 cells with GS-9620. (A) PBMCs were isolated from ART-suppressed HIV-infected individuals (*n* = 8) and treated in triplicate with GS-9620 or depleted of CD8 T cells and then treated with vehicle control (DMSO), 100 nM GS-9620, 1 mM GS-9620, or PMA and ionomycin. After 4 days, viral RNA was quantified in the cell culture supernatants. (B) CD4 cells were isolated from ART-suppressed HIV-infected individuals in whom GS-9620 induced HIV RNA at least 1.5-fold in PBMCs (*n* = 5) and treated in triplicate with vehicle, 100 nM GS-9620, or PMA and ionomycin. After 4 days, viral RNA was quantified in the cell culture supernatants. Significant differences from the vehicle (DMSO) control were determined by paired Student's *t* tests. *, *P* < 0.05; **, *P* < 0.01; ***, *P* < 0.001.

As TLR agonists activate antiviral immunity, it is possible that effector cells, such as CD8^+^ T cells, may limit virion production in PBMCs treated with GS-9620. To address this, we depleted CD8 T cells from PBMCs and activated them with GS-9620 as described above. Higher levels of virion production were consistently seen across all conditions tested when CD8 T cells were depleted from PBMCs (Fig. 2A). The geometric mean fold increase in GS-9620-treated samples relative to vehicle controls also increased in CD8-depleted PBMCs (1.5- and 2.2-fold at 100 nM and 1 μM GS-9620, respectively) relative to PBMCs containing CD8 T cells from the same individuals (1.4- and 1.6-fold at 100 nM and 1 μM GS-9620, respectively), but these increases were not significant. We also assessed the activity of GS-9620 with isolated CD4 T cells. Consistent with the low level of TLR7 expression previously found in CD4 T cells (22), GS-9620 had no effect on virion production from isolated CD4 T cells (Fig. 2B).

### IFN-α is strongly induced by GS-9620 in HIV-infected PBMCs.

**FIG 3:**
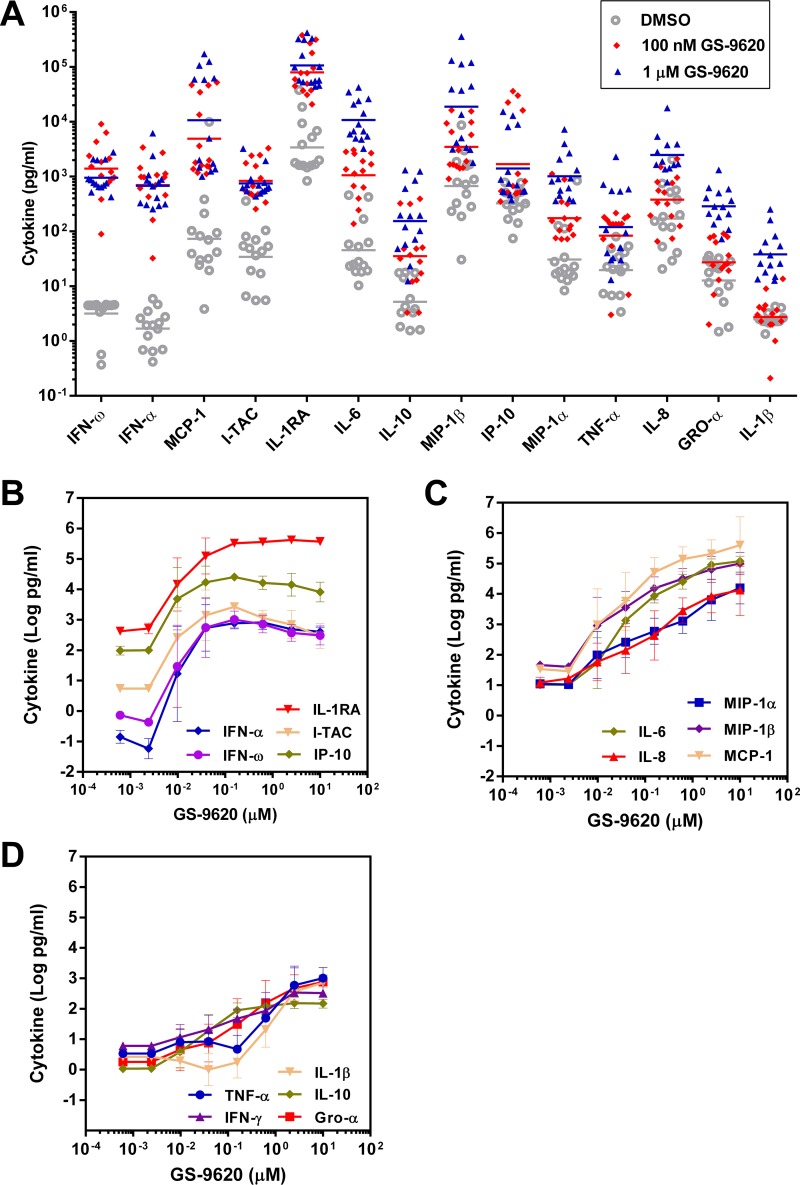
GS-9620 induces type I IFNs. Cytokine levels observed in cell culture supernatants after treatment of PBMCs isolated from HIV-infected individuals with the indicated concentrations of GS-9620. (A) Results from individual donor PBMC samples (*n* = 14) for select GS-9620 concentrations. The lines represent the geometric means. (B to D) GS-9620 dose responses for induced cytokines (*n* = 4). The data represent means of three replicates ± standard deviations.

GS-9620 was originally identified based on its ability to selectively induce IFN-α far more potently than tumor necrosis factor alpha (TNF-α) in PBMCs isolated from healthy donors (29). To further characterize the soluble factors secreted upon treatment of PBMCs from HIV-infected individuals, we used multiplex immunoassays to analyze cytokine and chemokine levels in culture supernatants 2 days after treatment with DMSO (vehicle control) or with 100 nM or 1 μM GS-9620. Relative to vehicle control cultures, GS-9620 potently induced over 300-fold increases in IFN-α and IFN-ω production with both GS-9620 doses tested (Fig. 3A). Several other proinflammatory cytokines and chemokines increased at least 5-fold with 100 nM GS-9620 treatment, including MCP-1, I-TAC, IL-1RA, IL-6, IL-10, MIP-1β, IP-10, and MIP-1α. These results are consistent with clinical studies in healthy individuals, where GS-9620 induced IFN-α, MCP-1, I-TAC, IL-1RA, IL-6, MIP-1β, and IP-10 (30). With the exceptions of IFN-α, IFN-ω, I-TAC, and IP-10, higher doses of GS-9620 induced greater cytokine production. TNF-α, IL-8, GRO-α, and IL-1β increased at least 5-fold only with the higher dose, 1 μM GS-9620 (Fig. 3A). Other cytokines and chemokines, including IFN-γ, IL-2, IL-4, IL-5, IL-12p70, IL-15, IL-21, IL-23, RANTES, SDF-1α, and TRAIL, were assessed but did not increase at least 5-fold with GS-9620 treatment (see Materials and Methods).

More extensive characterization of the dose responsiveness of cytokine production was performed in PBMCs from 4 donors (Fig. 3B). On average, IFN-α, IFN-ω, I-TAC, IL-1RA, and IP-10 reached the maximum attainable response with doses of less than 200 nM GS-9620. Among PBMCs from different donors, peak IFN-α production was reached between 39 and 625 nM GS-9620. To cover this range in responses, we used both 100 nM and 1 μM throughout the experiments described below. At higher doses of GS-9620, IFN-α production decreased so that 10 μM GS-9620 induced 3.4-fold less IFN-α than the peak production. IFN-ω, I-TAC, and IP-10 similarly decreased at doses greater than 200 nM GS-9620 (Fig. 3B). In contrast, 10 μM GS-9620 induced the highest level of production for the other analytes assessed (Fig. 3C and D).

### Induction of latency reversal by GS-9620 requires IFN-α production.

**FIG 4:**
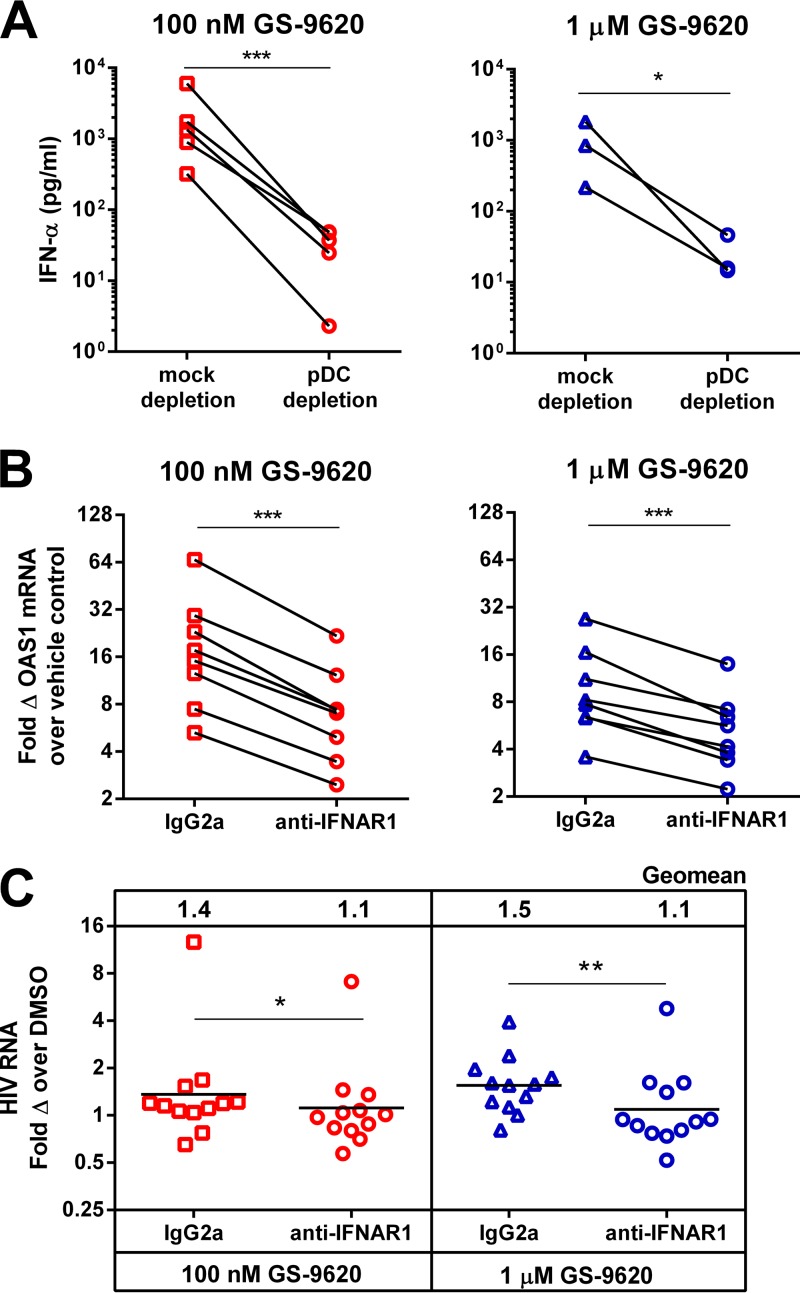
Activation of HIV RNA by GS-9620 is dependent on pDC-mediated production of type I IFNs. (A) PBMCs from ART-suppressed individuals were depleted of CD303^+^ CD304^+^ pDCs by magnetic beads prior to 100 nM GS-9620 (*n* = 5) or 1 μM GS-9620 (*n* = 3) treatment, and supernatant IFN-α levels were determined at 48 h from two replicates by Luminex. (B) Similar PBMCs were treated with anti-IFNAR1 monoclonal antibody or isotype control antibody (IgG2a) at the same time as GS-9620, and cellular OAS1 (*n* = 8) was measured in two replicates 24 h poststimulation by quantitative PCR. (C) Viral RNA was quantified in cell culture supernatants 4 days after treatment with GS-9620 and either anti-IFNAR1 or isotype control antibody (IgG2a). For viral RNA, each symbol represents the geometric mean of 12 replicates from a single individual. The horizontal bars indicate the geometric means of the 12 donors tested, which are also shown at the top of the graph. Significant differences from the vehicle (DMSO) control were determined by two-tailed paired Student's *t* tests. *, *P* < 0.05; **, *P* < 0.01; ***, *P* < 0.001.

GS-9620 is a potent agonist of TLR7, which is highly expressed on pDCs (34). In order to determine if pDCs play a role in the induction of HIV RNA with GS-9620, PBMCs were depleted of pDCs or mock depleted. On average, we depleted 65% of pDCs from PBMCs isolated from HIV-infected donors. This was lower than the 95% depletion we routinely achieved in PBMCs from HIV-negative donors using the same method but nonetheless resulted in significant reductions of IFN-α production in response to GS-9620 (Fig. 4A). These depletion experiments confirmed that pDCs from HIV-infected donors are the primary source of IFN-α induced by GS-9620, similar to what was previously reported for other TLR7 agonists in cells from healthy donors (35).

To determine if the induction of type I IFNs plays a role in the mechanism of HIV induction by GS-9620, an antibody to IFN-α/β receptor (anti-IFNAR1) was used to block type I IFNs from binding their receptor. Induction of the ISG OAS1 was significantly diminished in the presence of anti-IFNAR1 compared to isotype IgG2a (Fig. 4B). Similarly, anti-IFNAR1 decreased induction of ISG15 and Mx1. GS-9620 did not induce HIV RNA in the presence of anti-IFNAR1 (Fig. 4C). Treatment with anti-IFNAR1 led to decreased HIV RNA production relative to the isotype control in PBMCs from 9 of the 12 donors assessed at 1 μM GS-9620 (*P* = 0.0054). The median decrease with anti-IFNAR1 at 1 μM GS-9620 was 1.4-fold, with an interquartile range of 1.1- to 1.6-fold. Together, these results suggest that type I IFNs produced by pDCs are required for HIV RNA induction by GS-9620.

### GS-9620 enhances CD8 and CD4 T cell activation.

**FIG 5:**
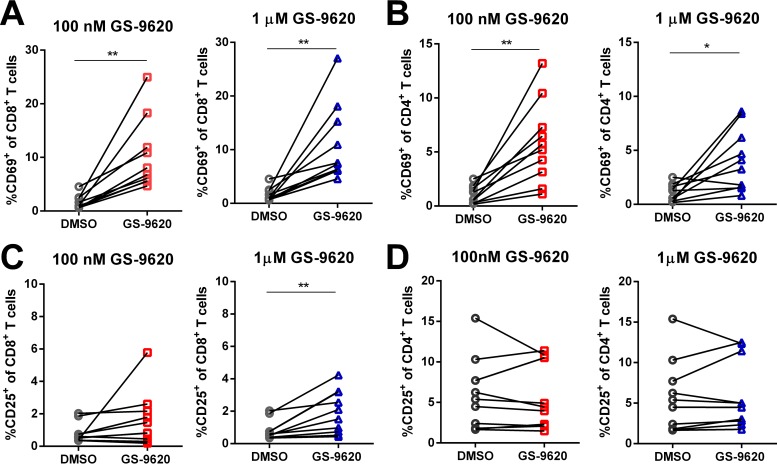
GS-9620 enhances CD69 expression on CD8^+^ and CD4^+^ T cells. PBMCs isolated from ART-suppressed HIV-infected individuals (*n* = 9) were measured for CD69 (A and B) or CD25 (C and D) activation on CD8^+^ (A and C) and CD4^+^ (B and D) T cells by flow cytometry after treatment with GS-9620 at the indicated concentrations. The *P* values were determined by paired Student's *t* tests. *, *P* < 0.05; **, *P* < 0.01; ***, *P* < 0.001.

Preclinical studies in chimpanzees infected with hepatitis B virus have shown that GS-9620 upregulates CD69, a marker associated with T cell activation (25). To test if GS-9620 can similarly activate T cells from ART-suppressed HIV-infected individuals, we quantified CD69 expression on T cells following treatment with 100 nM or 1 μM GS-9620. In this context, CD69 was consistently upregulated on both CD8 (Fig. 5A) and CD4 (Fig. 5B) T cells with both tested concentrations of GS-9620 relative to DMSO vehicle control treatment. GS-9620 generally had much less effect on CD25 expression on CD8 (Fig. 5C) and CD4 (Fig. 5D) T cells. The lack of CD25 activation was consistent with the lack of IL-2 stimulation mentioned above.

The stimulation of virus production from latently infected cells during TLR activation may enhance the presentation of viral antigens and the HIV-specific immune response. To assess the effect of GS-9620 on HIV-specific T cell activation, we labeled PBMCs with carboxyfluorescein succinimidyl ester (CFSE) and stimulated them with a pool of HIV peptides (derived from Gag, Pol, Env, and Nef) in the presence or absence of GS-9620. After 8 days, a recall response was elicited by repeated treatment of PBMCs with the same pool of HIV peptides or control peptides derived from cytomegalovirus (CMV)/Epstein-Barr virus (EBV)/influenza (flu) virus/tetanus bacterium (CEFT). The second stimulations with control (CEFT) peptides were included to determine if the proliferating cells were specifically responding to HIV antigen stimulation or were proliferating nonspecifically. Subsequently, CD8 and CD4 T cells were stained for intracellular production of IFN-γ and TNF-α and for surface expression of CD107a, indicating degranulation (36).

**FIG 6:**
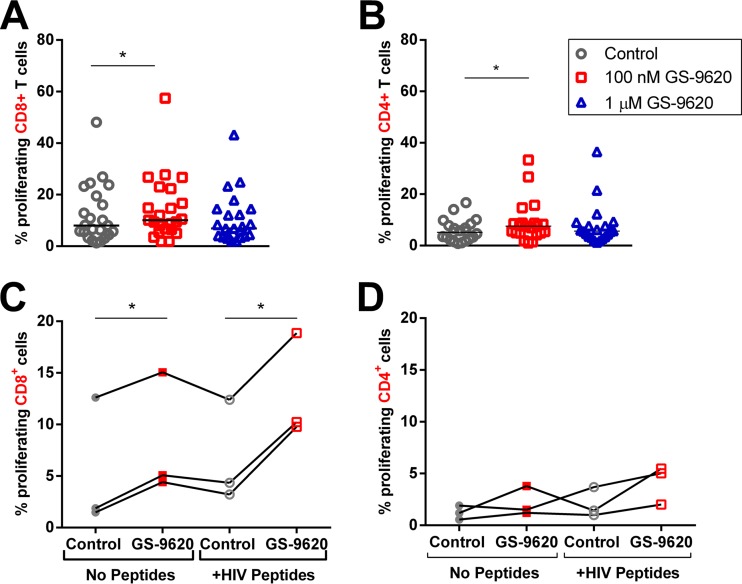
GS-9620 enhances proliferation of HIV-specific CD8^+^ and CD4^+^ T cells. CFSE-labeled PBMCs were isolated from ART-suppressed HIV-infected individuals (*n* = 23) and stimulated in duplicate with a pool of HIV peptides (derived from Gag, Nef, Env, and Pol) with or without GS-9620 for 8 days. The cells were treated a second time with the same HIV peptides, stained for activation, and analyzed by flow cytometry. (A) Scatter plots showing the percentages of proliferating (CFSE dim) CD8^+^ and CD4^+^ T cells. The cells were treated with vehicle control, 100 nM GS-9620, or 1 μM GS-9620. Each symbol corresponds to the data from a single subject, with the horizontal bars indicating medians. (B) For a subset of CD8 and CD4 cells from individuals that proliferated in response to GS-9620 and HIV peptides, the cells were similarly treated, but without HIV peptide stimulation. The lines connect results obtained from the same individual. The *P* values were determined by paired Student's *t* tests. *, *P* < 0.05.

The overall levels of proliferating (CFSE dim) CD8 and CD4 T cells increased 1.3- and 1.5-fold, respectively, at 100 nM GS-9620 but did not significantly change at 1 μM GS-9620 (Fig. 6A and B). To determine if this increase required both GS-9620 and antigenic stimulation, cells were also treated with GS-9620 in the absence of HIV peptides. In CD8 T cells (Fig. 6C), 100 nM GS-9620 significantly increased proliferation in both the presence (2.4-fold average increase) and absence (1.5-fold average increase) of antigen. The trends were similar but not significant in CD4 T cells (Fig. 6D). This indicates that activation by GS-9620 can occur independently of antigen specificity and is consistent with the CD69 results described above, which occur in the absence of antigen and at a rate higher than would be expected if only HIV-specific cells were activated. In both CD8 and CD4 cells from each individual assessed, the greatest proliferation was seen with both GS-9620 and HIV antigen.

**FIG 7:**
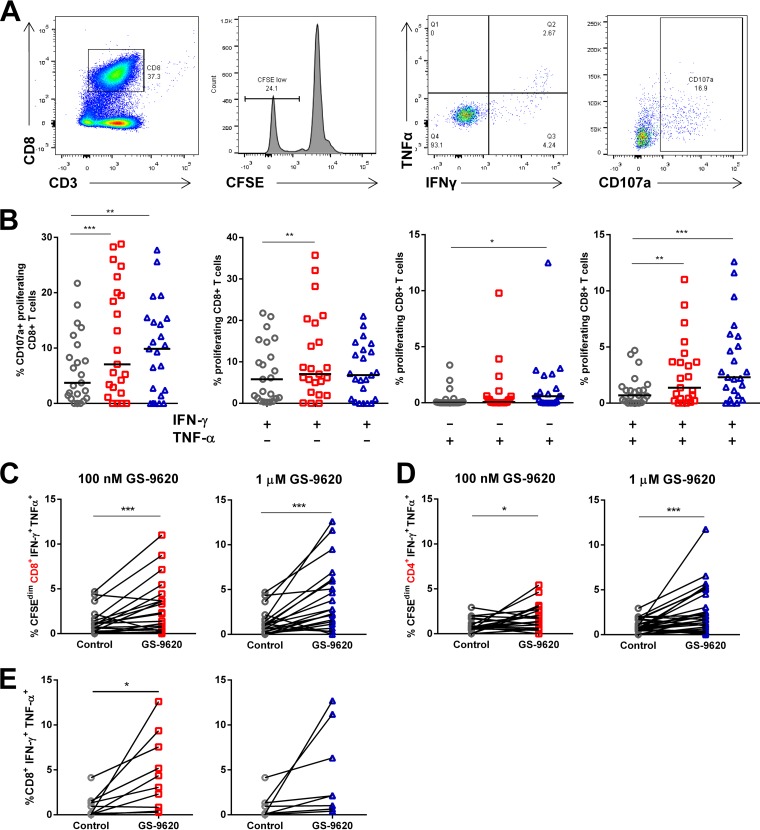
GS-9620 enhances polyfunctionality of HIV-specific CD8^+^ and CD4^+^ T cells. CFSE-labeled PBMCs were isolated from ART-suppressed HIV-infected individuals (*n* = 23) and stimulated with a pool of HIV peptides (derived from Gag, Nef, Env, and Pol) with or without GS-9620 for 8 days. The cells were then treated a second time with the same HIV peptides, stained for activation, and analyzed by flow cytometry. (A) Representative gating for proliferating (CFSE low) CD8^+^ T cells positive for CD107a or IFN-γ and TNF-α. (B) Scatter plots showing the percentages of proliferating (CFSE dim) CD8^+^ and CD4^+^ T cells expressing CD107a, IFN-γ, TNF-α, or IFN-γ and TNF-α. The cells were treated with vehicle control (circles), 100 nM GS-9620 (squares), or 1 μM GS-9620 (triangles). Each symbol represents data from a single individual, with the horizontal bars indicating medians. (C and D) Intracellular cytokine staining was used to assess production of IFN-γ and TNF-α in proliferating CD8^+^ (C) and CD4^+^ (D) T cells compared to vehicle control-treated samples. The lines connect values from the same individual. (E) CFSE-labeled PBMCs were isolated from healthy donors and stimulated with a pool of common antigenic (CEFT) peptides with or without GS-9620 for 8 days. The cells were then treated a second time with the same CEFT peptides, stained for activation, and analyzed by flow cytometry. The cells were treated with vehicle control (circles), 100 nM GS-9620 (squares; *n* = 10), or 1 μM GS-9620 (triangles; *n* = 8). The *P* values were determined by paired Student's *t* tests. *, *P* < 0.05; **, *P* < 0.01; ***, *P* < 0.001.

To determine if GS-9620 increased CD8 and CD4 T cell function, we gated on markers associated with cytotoxicity, including CD107a and intracellular IFN-γ and TNF-α (representative gating schemes are shown in Fig. 7A). Stimulation with GS-9620 led to significant increases in each of these markers in proliferating (CFSE dim) CD8 T cells (Fig. 7B and C). The greatest response to GS-9620 was seen in polyfunctional proliferating CD8 T cells that produced both IFN-γ and TNF-α. This population increased 2.0-fold with 100 nM GS-9620 (*P* = 0.0034) and 3.3-fold with 1 μM GS-9620 (*P* = 0.00057) compared to the control treatment. Increases were seen in PBMCs from 18 of 23 donors with 1 μM GS-9620 (Fig. 7C). Similarly, proliferating CD4 T cells making both IFN-γ and TNF-α increased 1.9-fold at 100 nM (*P* = 0.019) and 2.3-fold at 1 μM (*P* = 0.00089) GS-9620. Increases were seen in PBMCs from 22 of 31 donors with 1 μM GS-9620 in CD4^+^ T cells (Fig. 7D).

Similar experiments were performed in PBMCs from healthy individuals that were initially stimulated with CEFT peptides and then recalled with CEFT peptides, as well (Fig. 7E). Similar to the results seen with response to HIV antigen, the level of proliferating CD8 T cells producing IFN-γ and TNF-α increased 7-fold with 100 nM GS-9620 (*P* = 0.018) and 3.9-fold with 1 μM GS-9620 (*P* = 0.077). These results show that GS-9620 is able to stimulate T cells independently of antigen specificity.

**FIG 8:**
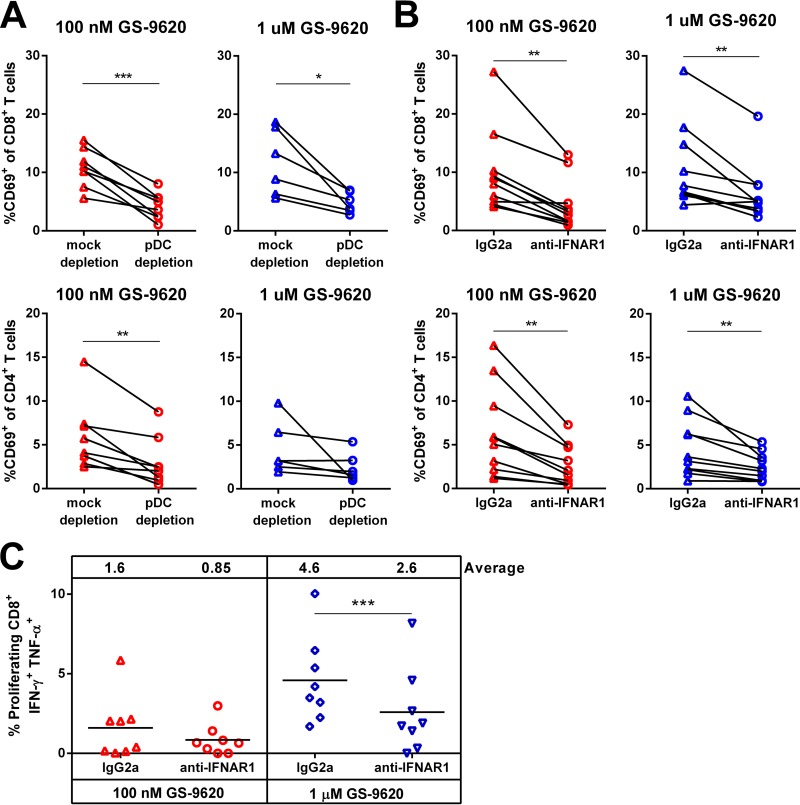
pDCs and type I IFNs are required for T cell activation by GS-9620. (A and B) PBMCs isolated from ART-suppressed HIV-infected individuals and stimulated with GS-9620 were depleted of pDCs prior to stimulation (A) or treated with anti-IFNAR1 at the time of stimulation where noted (B). Flow cytometry was used to measure CD69 levels in CD8^+^ T cells and CD4^+^ T cells 48 h after GS-9620 treatment. (C) CFSE-labeled PBMCs were stimulated with a pool of HIV peptides (Gag, Pol, Env, and Nef) with or without GS-9620 for 8 days. The cells were restimulated with HIV peptides and stained for intracellular cytokines. The horizontal bars and the numbers at the top of each graph indicate the average percentages of IFN-γ^+^ TNF-α^+^ cells among proliferating cells, with each symbol representing a PBMC sample from a single donor. The *P* values were determined by paired Student *t* tests. *, *P* < 0.05; **, *P* < 0.01; ***, *P* < 0.001.

To determine if pDC-mediated production of type I IFNs plays a role in enhancing effector function, T cell activation was assessed in the context of pDC depletion or type I IFN blockade. Similar to the preceding experiments, pDC depletion reduced IFN-α production and anti-IFNAR1 decreased expression of ISGs. T cell activation, as determined by CD69 upregulation, was directly dependent on both pDCs (Fig. 8A) and type I IFN (Fig. 8B). This dependence was most evident in CD8 T cells, where increased CD69 was most prominent, but significant differences were also seen with CD4 T cells treated with 100 nM GS-9620. In the presence of the anti-IFNAR1 monoclonal antibody (MAb), proliferating CD8 T cells produced significantly less IFN-γ and TNF-α in response to GS-9620 stimulation than with isotype MAb control (Fig. 8C). The anti-IFNAR1 MAb decreased the number of proliferating CD8 T cells producing IFN-γ and TNF-α relative to the isotype control in every donor stimulated with 1 μM GS-9620 and in 7 of 8 donors stimulated with 100 nM GS-9620. Some activation was seen at 1 μM GS-9620 even in the presence of anti-IFNAR1 antibody, indicating that additional signals produced at this concentration also stimulate CD8 T cells. These results clearly indicate that type I IFNs produced by TLR7-stimulated pDCs contribute to the activation of T cells and the enhancement of their immune effector function.

### Treatment with GS-9620 increases CD8 T cell cytolytic activity.

In order to assess the cytolytic activity of CD8 T cells after treatment with GS-9620, we measured the induction of active caspase 3 in target cells. Granzyme delivery into target cells triggers caspase 3 cleavage and activation, leading to apoptosis and death. Previous work has shown that assays detecting activated caspase 3 in target cells yield results comparable to traditional ^51^Cr release assays but with higher sensitivity (37, 38).

**FIG 9:**
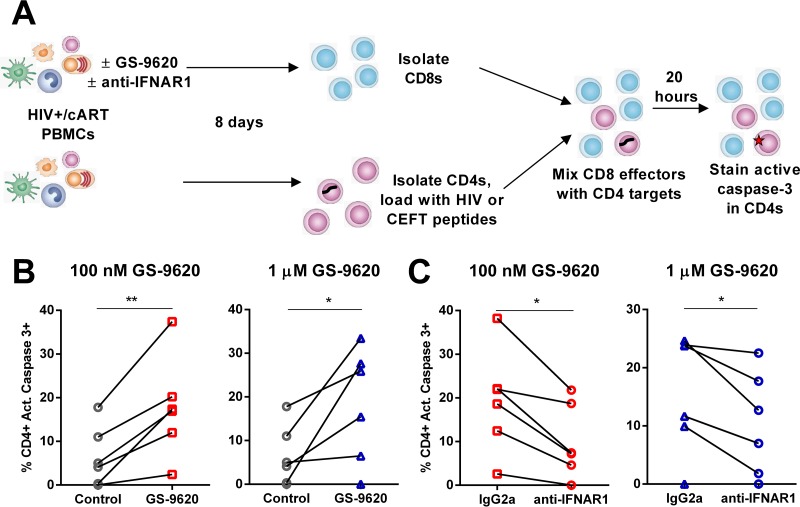
Polyfunctional CD8^+^ T cells generated by GS-9620 exhibit cytotoxic activity. (A) PBMCs isolated from ART-suppressed HIV-infected individuals (*n* = 6) were stimulated with a pool of HIV peptides (Gag, Pol, Env, and Nef) with or without the indicated concentrations of GS-9620 for 8 days before assessment of killing capacity. Either anti-IFNAR1 or isotype IgG2a antibody was added to the cultures. CD8^+^ T cells were isolated and cocultured with autologous CD4^+^ T cells pulsed with HIV peptides for 20 h at an effector/target ratio of 5:1. (B) CD4^+^ T cells pulsed with control peptides were used to assess nonspecific killing of cells that did not display HIV antigen. After 20 h of coculture, the cells were stained for active caspase 3 in target CD4^+^ T cells and analyzed by flow cytometry. Shown are the percentages of HIV-specific CD4^+^ T cells expressing active caspase 3. HIV-specific values were obtained by subtracting the corresponding percentage of marker-positive cells in control peptide-pulsed targets from HIV peptide-pulsed targets. (C) Cells were treated with anti-IFNAR1 or isotype control antibody at the same time as GS-9620 and maintained throughout the 8 days of stimulation. Each symbol indicates data from a single subject. The *P* values were determined by paired Student's *t* tests. *, *P* < 0.05; **, *P* < 0.01.

PBMCs from ART-suppressed HIV-infected individuals (*n* = 6) were stimulated with a pool of HIV peptides (derived from Gag, Pol, Env, and Nef) in the presence or absence of GS-9620. Where specified, either anti-IFNAR1 or isotype IgG2a antibody was added to the stimulated cultures. After 8 days, CD8 T cells were isolated from these cultures. In parallel, unstimulated autologous PBMC cultures were also maintained for 8 days, and CD4^+^ T cells were isolated. The CD4 T cells were then loaded with either HIV peptides or control CEFT peptides to generate target cells and mixed with isolated effector CD8 T cells at an effector/target ratio of 5:1 (Fig. 9A). Coculture of the effector and target cells in the presence of either 100 nM or 1 μM GS-9620 significantly increased the percentage of HIV antigen-loaded target cells that stained positive for cleaved caspase 3 from 6.4% in the vehicle-treated control to 18% at both 100 nM and 1 μM GS-9620 (*P* = 0.0069 at 100 nM and *P* = 0.030 at 1 μM GS-9620). In contrast, 14% of CEFT-loaded target cells stained positive for cleaved caspase 3, and this did not vary more than 1% across the conditions assessed. GS-9620 increased apoptosis in target cells in all 6 samples treated with 100 nM and in 5 of 6 samples treated with 1 μM GS-9620 (Fig. 9B).

Activation of cytolytic activity clearly required type I IFNs, as treatment with the anti-IFNAR1 antibody significantly decreased GS-9620-dependent cytolytic activity relative to cultures treated with the isotype control antibody (Fig. 9C). Average levels of apoptotic targets decreased from 19% in the isotype-treated samples to 10% under the anti-IFNAR1-treated conditions at 100 nM and from 16% to 10% at 1 μM GS-9620 (*P* = 0.011 at 100 nM and *P* = 0.030 at 1 μM GS-9620). Parallel CD8 cultures from the same donor PBMCs also showed that CD8 cells that proliferated and produced intracellular IFN-γ and TNF-α in response to HIV peptides increased upon treatment with GS-9620 in a type I IFN-dependent manner (data not shown), consistent with the results described above. The increased levels of polyfunctional CD8 T cells were consistent with the elevation of HIV-specific cytolytic activity. GS-9620 clearly activated CD8 T cells and enhanced killing of CD4 cells displaying HIV peptides through a mechanism that required type I IFNs.

### Antibody-dependent killing of HIV-infected cells is enhanced by GS-9620.

Several immune mechanisms can mediate clearance of latently infected CD4 T cells. In addition to T cell-mediated cytotoxicity, HIV-infected cells can be targeted by antibody-dependent cellular cytotoxicity or phagocytosis. Broadly neutralizing antibodies (bNAbs) have shown promise in preclinical models based on their ability to induce killing of HIV-infected cells through these mechanisms (12, 39).

**FIG 10:**
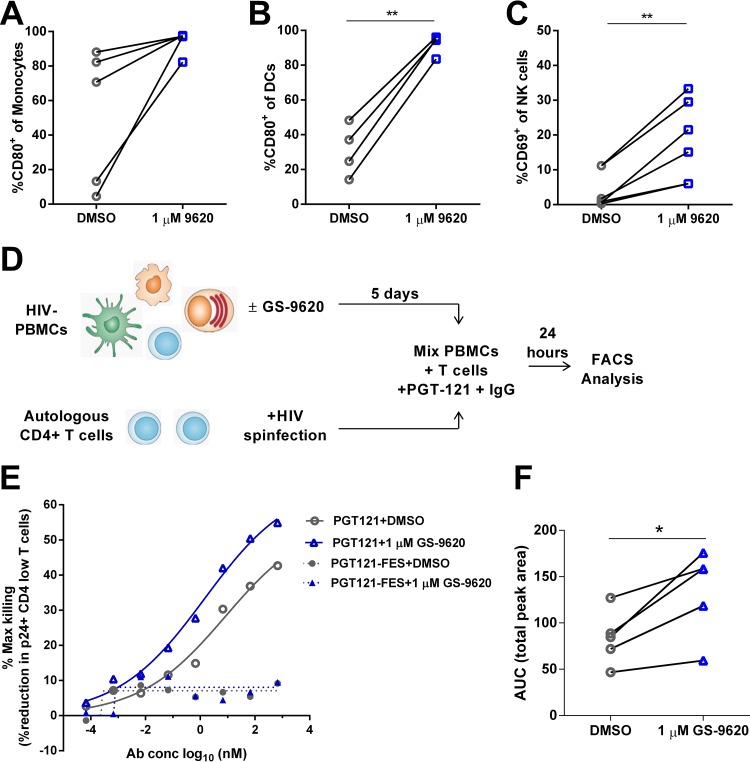
(A to C) GS-9620 activates immune effector cells and enhances PGT121 antibody-mediated killing of HIV^+^ CD4 cells. PBMCs from healthy donors were treated with 1 μM GS-9620 and stained for CD69 or CD80 expression on monocytes (*n* = 5) (A), DCs (*n* = 4) (B), and NK cells (*n* = 6) (C). (D) Schematic describing the antibody-mediated killing assay using GS-9620-treated PBMCs as effectors. Briefly, PBMCs from healthy donors were treated with 1 μM GS-9620 or DMSO and cultured for 5 days. Simultaneously, autologous donor CD4 T cells were isolated, spinfected with HIV-1 BaL, and cultured at 37°C for 5 days. On day 5, the HIV-infected CD4 T cells were mixed with titrations of PGT121 antibody and human IgG. After opsonization for 30 min, GS-9620- or DMSO-treated PBMCs were mixed in at an effector/target ratio of 20 PBMCs to 1 infected CD4 cell and incubated at 37°C overnight. The next day, the cells were stained for p24, and HIV-infected cells were quantified by fluorescence-activated cell sorter (FACS). (E) Average values from 5 donors. (F) Peak AUC from results plotted as in panel E were compared for DMSO-treated and GS-9620-treated PBMCs from each individual donor. The *P* values were determined by paired Student's *t* tests. *, *P* < 0.05; **, *P* < 0.01.

Previous work suggested that TLR7 agonists can induce the maturation and activation of the effector cells required for antibody-mediated killing (25, 40). In PBMCs from HIV-infected individuals, we found that GS-9620 upregulated the maturation marker CD80 on both monocytes and dendritic cells (Fig. 10A and B). Analysis of NK cells in these cultures showed upregulation of the activation marker CD69 on this effector cell type, as well (Fig. 10C).

To determine if activation of immune effector cells by GS-9620 could enhance antibody-mediated killing (Fig. 10D), PBMCs were treated with GS-9620 for 5 days and combined for 24 h with target CD4 T cells and various concentrations of PGT121, a broadly neutralizing antibody that has been used in a nonhuman primate model to target latent virus (39). A resting, *in vitro* CD4 T cell model of HIV infection was used to mimic the resting state of latently infected cells *ex vivo* (41–43). Elimination of target cells was measured by p24 staining 24 h after coculture by flow cytometry. An improvement in the maximal killing of HIV-infected target cells, as well as in the potency of killing, was seen when PBMCs were pretreated with GS-9620 (Fig. 10E). We introduced L234F/L235E/P331S (FES) mutations that disrupt Fc receptor binding into PGT121 to determine if this killing was dependent on effector function (44). No killing was seen with the FES version of PGT121. In PBMCs from all donors tested (*n* = 5), the area under the dose-response killing curve (AUC) increased when effector cells were prestimulated with GS-9620 (Fig. 10F). Taken together, these results indicate that immune effector cells exposed to GS-9620 more effectively recognize and kill HIV-infected target cells in the presence of effector-competent bNAbs.

## DISCUSSION

In PBMCs isolated from ART-suppressed HIV-infected donors, we showed that GS-9620 can both stimulate HIV expression and enhance immune functions relevant for targeting the viral reservoir. GS-9620 activated type I immunity by stimulating cytokine production and upregulating established activation markers on T cells, NK cells, macrophages, and dendritic cells. Specifically, in CD8 T cells, treatment with GS-9620 concordant with HIV antigen stimulation increased degranulation, IFN-γ production, and TNF-α production, each of which is associated with cytolytic activity. This correlated with a greater ability to induce apoptosis in CD4^+^ T cell targets. Similarly, GS-9620-treated PBMCs were more effective at clearing HIV-infected cells in the presence of an effector-competent bNAb, PGT121. These data suggest that GS-9620 may both increase HIV antigen expression in latently infected cells and stimulate their immune-mediated clearance by either innate or antigen-specific immune effector cells.

Our results showing that GS-9620 modestly increased HIV RNA are consistent with previous work indicating that TLRs can activate latent HIV (15–18). Much like TLR7, TLR9 is highly expressed on pDCs and B cells and induces high levels of type I IFN production (19–22). Offersen and colleagues found that a TLR9 agonist, MGN1703, increased HIV-1 unspliced RNA 1.4-fold in primary CD4 T cells that were activated in the context of PBMCs (16). Agonists of TLR8, which is expressed by monocytes and myeloid cells, increased HIV expression in U1 and OM-10 cell lines (15). Novis and colleagues tested a panel of TLR agonists in a primary CD4 T cell model of HIV latency and found that Pam3CSK4, a TLR1/2 agonist, induced HIV-1 transcription (17). In the latter study, agonists targeting TLRs that are not expressed at high levels in CD4 T cells (i.e., TLR7, TLR8, and TLR9) were inactive (17, 22). Collectively, these studies show that TLR agonists can induce HIV latency reversal and that this activity requires the presence of cell types that express sufficient levels of the targeted TLR. These results indicate that common downstream signaling pathways upregulated by multiple innate receptors may lead to latency reversal. Here, we extend these conclusions to show that a clinically tested TLR7 agonist induces HIV expression and that this induction is dependent on type I IFN production. This suggests that TLR9-mediated latency reversal may also be dependent on type I IFN production.

TLR7 agonists are reported to inhibit HIV replication through a mechanism that depends on type I IFNs (45). Several ISGs have been shown to inhibit steps late in the viral life cycle, potentially reducing virion production and infectivity (46). The moderate level of HIV induced may, in part, be the net response of CD4 T cells to the activating and inhibiting factors induced by type I IFNs, as well as other cytokines present in the pluripotent mixture induced by GS-9620.

pDCs are a key part of the innate immune system that responds to viral nucleic acids by producing type I IFN and other stimulatory cytokines (35). Upon stimulation, pDCs can devote up to 60% of their transcriptome to expressing type I and type III IFNs (47). This, combined with the low expression of TLR7 in T cells, led us to propose a model in which TLR7 activation by GS-9620 on pDCs leads to the production of cytokines that include type I IFNs that activate T cells. In support of this model, we found that depleting pDCs from PBMCs decreased both IFN-α production in response to TLR7 and CD69 upregulation on CD4 and CD8 T cells. To determine if soluble factors secreted by pDCs play a role in the activation of T cells from HIV-infected individuals as well, an IFNAR1-blocking antibody was used to selectively disrupt type I IFN signaling in treated cultures. This decreased GS-9620-mediated ISG induction in PBMCs and CD69 upregulation in CD4 and CD8 T cells. In addition, anti-IFNAR1 treatment neutralized the induction of polyfunctional CD8 T cells by GS-9620 and the cytotoxic activity of these cells. These data support our model that the induction of type I IFNs by GS-9620-treated pDCs is required for T cell activation through TLR7 stimulation.

An important challenge for the field of HIV research is to identify agents that activate latent virus but also have a safety profile compatible with repeated dosing in HIV-infected virally suppressed individuals. In our *ex vivo* PBMC assay, GS-9620 stimulated HIV RNA production in culture supernatants 1.5- to 2-fold. This magnitude of activation is lower than what has been reported after T cell activation by the mitogenic T cell activators phorbol myristate acetate (PMA) and ionomycin or anti-CD3/CD28 antibodies (48, 49). PKC agonists, such as bryostatin or ingenol B, achieve greater activation in cell culture (48, 49), but bryostatin has led to serious adverse events in oncology clinical trials (50–53). In HIV-infected individuals, tolerable doses of bryostatin had no effect on pharmacodynamic readouts associated with PKC activation, likely because they achieved only 5% of the systemic exposure that was predicted to be required for HIV latency reversal (54). In contrast, GS-9620 has been safely dosed in clinical studies with healthy and hepatitis B virus-infected individuals at levels that led to the ISG and cytokine induction associated with its activity (30, 55).

Repeated or intermittent doses of a well-tolerated agent with modest latency reversal activity for an extended period may be preferable to an agent predicted to induce greater latency reversal but also associated with more severe toxicity. It is difficult to model repeated TLR7 activation *ex vivo* because the responsiveness of pDCs to TLR7 stimulation is significantly altered by time after venipuncture (56). Assays done with PBMCs *in vitro* are also limited in their ability to reproduce the conditions encountered by memory T cells, which primarily reside in lymph nodes. However, recent results in ART-suppressed simian immunodeficiency virus (SIV)-infected rhesus macaques demonstrate that administration of a TLR7 agonist can induce transient increases in plasma SIV RNA *in vivo* and that repeated dosing improves this induction (57).

A therapeutically useful latency reversal agent should be able to induce HIV antigen expression without compromising the immune effector functions required to target activated reservoirs. Recent reports suggest that latency reversal agents, such as certain HDAC inhibitors, may impede CD8-mediated targeting of latently infected cells (58–61). In contrast, GS-9620 heightened cytotoxic effector function, as evidenced by elevated intracellular cytokine production in CD8 T cells and apoptosis induction in target CD4 T cells. This suggests that these effector cells are better able to target infected cells after HIV expression is induced. An obstacle to this is viral escape from cytotoxic T lymphocyte pressure through mutations in immunodominant epitopes (62). To circumvent this barrier, it may be useful to combine GS-9620 with a therapeutic vaccine that increases the breadth of the immune response and directs cytotoxic T lymphocytes to conserved unaltered epitopes (63).

In addition to activating T cell immunity, TLR7 agonists have been reported to enhance humoral immunity (64). We found that GS-9620 can induce activation of NK cells and phagocytic cells, which are capable of targeting infected cells for elimination through either cytotoxic activity or phagocytosis. Effector-competent antibodies can be used to recruit these immune effectors to latently infected cells in a highly specific manner (65, 66). To assess this, we used the PGT121 antibody, which has demonstrated antiviral effects *in vivo* and is a promising candidate for targeting the latent reservoir clinically (39). GS-9620 was used to activate the effector cells prior to coincubating them with latently infected target cells. The activation induced by GS-9620 improved the effectiveness of PGT121, increasing its potency and enhancing overall killing.

Together, these data demonstrate that GS-9620 can induce HIV expression in cells isolated from individuals on suppressive ART while also stimulating diverse effectors that can mediate the elimination of HIV-infected cells. Safety data show that GS-9620 is well tolerated in both healthy human subjects and individuals with chronic hepatitis B infection at doses inducing pharmacodynamic responses. The results from our *ex vivo* studies combined with the known clinical profile of GS-9620 support further assessment of GS-9620 for immune-mediated clearance of the latent HIV reservoirs in HIV-infected individuals on suppressive ART. Ongoing preclinical studies are exploring combinations of GS-9620 with other immune-based therapies in HIV latency models.

## MATERIALS AND METHODS

### Ethics statement.

HIV-infected participants were enrolled at Quest Clinical Research in San Francisco, CA. The study was approved by the Western Institutional Review Board. Informed written consent was obtained from participants before any study procedures were performed.

### Participants and cell isolation.

HIV-infected participants were selected based on sustained plasma viral load suppression (<50 copies/ml for at least 12 months), CD4 cell counts (>350 cells/μl), and absence of coinfection with hepatitis B or C virus. Clinical laboratory results were confirmed 2 weeks prior to leukapheresis or blood draw. Leukopaks were delivered and processed within 2 h postdraw. PBMCs were isolated by diluting the leukapheresis product 1:1 with PBS and layered over Ficoll. The cells were treated with red blood cell lysis buffer (eBioscience) and resuspended in complete RPMI medium (containing 10% fetal bovine serum [FBS] and penicillin-streptomycin), 100 nM elvitegravir, and 100 nM efavirenz to prevent new rounds of infection. For the experiments where CD8 T cells were depleted, the EasySep Human CD8 Positive Selection kit (Stem Cell) was used according to the manufacturer's instructions with the modification that beads bound to CD8 T cells were discarded and the remaining PBMCs were used as described below. For experiments where isolated CD4 T cells were used, the EasySep Human CD4^+^ T Cell Enrichment kit (Stem Cell) was used according to the manufacturer's instructions.

### Activation of HIV transcription from natural reservoirs *ex vivo*.

Fifteen million PBMCs or PBMCs depleted of CD8 T cells were added to wells of 6-well plates in 5 ml of RPMI medium with elvitegravir and efavirenz. For experiments with CD4 T cells, 5 million isolated CD4 T cells were added to wells of 24-well plates in 2.5 ml of RPMI medium with elvitegravir and efavirenz. The cells were stimulated with DMSO (vehicle control) or the indicated concentrations of GS-9620 immediately after cell isolation and incubated at 37°C. Anti-human IFNAR1 antibody (PBL Bioscience) or IgG2a isotype antibody (BD Bioscience) was added concurrently with GS-9620 at 10 ng/ml to specified cultures. After 4 days of incubation, 1 ml of supernatant from each well was collected and run on Cobas AmpliPrep/AmpliTaq for quantification of HIV-1 RNA using the HIV-1 test v2.0 kit (Roche Diagnostics). Eleven of 63 donors were excluded because their HIV RNA levels did not increase under the positive-control conditions (<5-fold increase in PMA and ionomycin versus vehicle-treated controls).

### ISG expression analyses.

After 24 h in culture, 3 × 10^5^ PBMCs were removed from each well and lysed with RLT lysis buffer (Qiagen). The cell lysates were stored at −80°C until they were ready for RNA isolation. RNA was then prepared from the cell lysates using an RNeasy 96-well kit (Qiagen). A TaqMan RNA-to-Ct One-Step kit (Applied Biosystems) was used with primers and probes to OAS1 (Hs00242943_m1), ISG15 (Hs01921425_s1), MX1 (Rh02842279_m1), and β-actin (HS00357333_g1) (Thermo Fisher Scientific). Transcript abundance was normalized to β-actin expression using 2^−[*Ct*(target gene) − *Ct*(β-actin)]^ and reported as fold change relative to the control conditions.

### Cytokine/chemokine analysis.

After 48 h in culture, supernatants were collected from each well and stored at −80°C until they were ready for analysis. The supernatants were inactivated using 10% Triton X and probed, using the ProcartaPlex multiplex immunoassay (Affymetrix eBioscience) according to the manufacturer's instructions, for 29 cytokines and chemokines: APO-FAS, BAFF, granzyme B, GRO-α, IFN-α, IFN-γ, IFN-ω, IL-1β, IL-1RA, IL-10, IL-12p70, IL-15, IL-2, IL-21, IL-23, IL-27, IL-29, IL-6, IL-8, IL-10, I-TAC, MCP-1, MIP-1α, MIP-1β, MMP-1, RANTES, SDF-1α, TNF-α, and TRAIL. Samples were then read on a Luminex 200 (Luminex) platform and analyzed using Bio-plex Manager software (Bio-Rad). Previous analyses were also done with a multiplex that included IL-4, IL-5, IL-7, IL-13, IL-17A, IL-18, IL-31, and sCD-40L, but they were found to be unaffected by GS-9620 treatment.

### Measurement of immune cell activation.

After 48 h in culture, PBMCs were removed from each well and stained with LIVE/DEAD Fixable Aqua dead cell stain (Thermo Fisher Scientific). T cells (CD3^+^) were stained with V450-labeled antibody to CD4, Alexa Fluor-700-labeled antibody to CD3, allophycocyanin (APC)-H7-labeled antibody to CD8, phycoerythrin (PE)-labeled antibody to CD69, and APC-labeled antibody to CD25 (BD Biosciences). NK cells (CD56^+^ CD16^−^ and CD56 dim CD16^+^) were stained with PE-Cy7-labeled antibody to CD56, PE-labeled antibody to CD16, and fluorescein isothiocyanate (FITC)-labeled antibody to CD69 (BD Biosciences). To assess effects on monocytes and dendritic cells, adherent cells were detached using cold phosphate-buffered saline (PBS) containing 1 mM EDTA prior to LIVE/DEAD staining. Monocytes (CD16^+^) were stained with PE-labeled antibody to CD16, AF700-labeled antibody to CD69, and BV650-labeled antibody to CD80 (BD Biosciences). Dendritic cells (HLA-DR^+^ Lineage^−^) were stained with APC-labeled antibody to HLA-DR, FITC-labeled antibodies to human lineage markers (CD3, CD16, CD19, CD20, CD14, CD56), BV421-labeled antibody to CD69, and BV650-labeled antibody to CD80 (BD Biosciences). The cells were treated with fixation buffer (eBioscience) and analyzed using an LSR Fortessa (BD Biosciences) and FlowJo software (Treestar).

### pDC depletion.

PBMCs were resuspended at 1 × 10^8^ cells/ml in isolation buffer (PBS plus 2% FBS and 1 mM EDTA) with 100 μl/ml each of biotin-labeled antibody to CD303 (Miltenyi Biotec), biotin-labeled antibody to CD304 (Miltenyi Biotec), and FcR block (Stem Cell Technologies). For mock-depleted samples, only 100 μl/ml of FcR block was added. Biotin-labeled cells and mock-labeled cells were then isolated using an EasySep Biotin Positive Selection kit (Stem Cell Technologies). The cells were resuspended in medium and stimulated as described above. The purity of depletion was assessed by staining freshly isolated cells with FITC-labeled antibodies to human lineage markers, peridinin chlorophyll protein (PerCP)-Cy5.5-labeled antibody to CD123, APC-labeled antibody to CD11c, and V450-labeled antibody to HLA-DR (BD Biosciences) for 15 min. The cells were treated with fixation buffer (eBioscience) and analyzed on an LSR Fortessa (BD Biosciences) with FlowJo software (Treestar).

### HIV-specific T cell expansion assay.

PBMCs were prepared as described above from ART-suppressed HIV-infected individuals or healthy individuals, as indicated, and labeled with 0.1 μM Cell Trace CFSE (Thermo Fisher Scientific). The cells were then treated with the indicated GS-9620 concentrations, 500 ng/ml of each HIV peptide pool (Env, Pol, Gag, and Nef; PepMix HIV Ultra pools; JPT) or 500 ng/ml of a pool of peptides derived from common antigens (CEFT; JPT), 50 U/ml IL-2, 100 nM efavirenz, and 100 nM elvitegravir. Anti-human IFNAR1 (PBL Bioscience) or anti-mouse IgG2a isotype (BD Bioscience) was added concurrently with GS-9620 at 10 ng/ml to specified cultures. After 8 days of incubation, the cells were washed with medium twice. Each stimulated well was split into two replicates: one set was restimulated with 500 ng/ml of the same HIV peptide pool, and the other set was recalled with 500 ng/ml of CEFT peptide (JPT Peptides). Both sets were treated with PE-Cy7-labeled antibody to CD107a (BD Biosciences) and incubated for 1 h at 37°C. Monensin and brefeldin A (BD Biosciences) were added to each well and incubated for an additional 3 h. The cells were then stained with LIVE/DEAD Fixable Aqua dead cell stain (Thermo Fisher Scientific) for 15 min. The cells were quenched with medium and stained for CD3, CD4, and CD8 as described above. The cells were fixed and permeabilized with an intracellular fixation and permeabilization buffer set (eBioscience) and then stained with APC-labeled antibody to IFN-γ (BD Bioscience) and PerCP-Cy5.5-labeled antibody to TNF-α (eBioscience). Samples were analyzed by flow cytometry using an LSR Fortessa (BD Biosciences) and FlowJo software (Treestar). To focus on the HIV-specific T cell response, values for cells that had been recalled with CEFT peptide were subtracted from those of cells treated with HIV peptides.

### HIV-specific T cell cytotoxicity assay.

PBMCs were treated with GS-9620, HIV peptide pools, IL-2, efavirenz, elvitegravir, and anti-IFNAR1 or isotype control as described above. Cultures were maintained for 8 days with a medium change on day 4, maintaining the HIV peptides and antibody concentrations. CD8 T cells were isolated by EasySep negative selection (Stem Cell; catalog no 19053) and used as effector cells.

Total CD4 T cells were isolated by negative selection (EasySep human CD4^+^ T cell enrichment; Stem Cell) from parallel, untreated cultures of autologous PBMCs and maintained in complete RPMI medium with IL-2 for 8 days, with medium change on day 4. Isolated CD4 T cells were pulsed with either HIV peptide pools (Gag, Nef, Pol, and Env pools; PepMix HIV Ultra pools; JPT) or a CEFT peptide pool (negative control) by incubating CD4 T cells at 10^6^ cells/ml with 1 μg/ml of each peptide pool in complete RPMI medium at 37°C for 1 h and then washing and resuspending them in medium.

Peptide-pulsed CD4 T cells (targets) were cocultured with CD8 T cells from various treatment conditions (effectors) at an effector/target ratio of 5:1 for 20 h in a V-bottom, 96-well tissue culture plate (CD4^+^ T cells were maintained at 200,000 cells/well in a 200-μl culture volume). After incubation, the cells were surface stained for CD4, CD8, and CD3 as described above. The cells were fixed and permeabilized using Cytofix/Cytoperm (BD Biosciences), stained with AF-647-labeled antibody to active caspase 3 (BD Biosciences), and analyzed by flow cytometry using an LSR Fortessa (BD Biosciences) and FlowJo software (TreeStar). HIV-specific CTL activity was determined by gating on CD4^+^ CD3^+^ cells with active caspase 3 and subtracting values with CEFT peptide-pulsed targets from HIV peptide-pulsed targets. Surface CD107a and intracellular IFN-γ and TNF-α levels were assessed in parallel aliquots from these cultures, using intracellular Ki67 to measure proliferation in CD8 T cells.

### Antibody-mediated killing of HIV-infected cells.

PBMCs were treated with 1 μM GS-9620 or DMSO (control) and cultured at 3 × 10^6^ cells/ml at 37°C for 5 days for use as effector cells. Simultaneously, autologous donor CD4 T cells were isolated as described above and spinfected with HIV-1 BaL at 1,200 × *g* for 2 h. The cells were washed twice and cultured for 5 days in the presence of IL-2 (30 U/ml). On day 5, HIV-1-infected CD4 T cells were labeled with PKH67 green fluorescent membrane dye (Sigma) for 5 min in the dark. The dye was neutralized by adding 100% FBS. The cells were washed 4 times and resuspended in RPMI with penicillin and streptomycin and 10% low-IgG serum (Gibco). HIV-infected CD4 T cells were opsonized for 30 min at 37°C with the indicated concentrations of PGT121 or a control PGT121 with FES mutations in the Fc domain that abrogated Fc receptor binding on effector cells (44). Effector cells treated with GS-9620 or DMSO were washed twice and cocultured overnight with labeled, opsonized CD4 target cells at a PBMC effector/CD4 target ratio of 20:1. The cells were then stained with Fixable Aqua dead cell stain and labeled with antibody to CD4. The cells were fixed using Cytofix/Cytoperm (BD Biosciences) and stained with PE-labeled antibody to p24 (KC57; Beckman Coulter). Viable HIV-infected cells were quantified as PKH67 green fluorescent membrane dye-positive, CD4 low, p24^+^ cells and normalized to the control condition under which no PGT121 antibody was added.

## Supplementary Material

Supplemental material
