# Supplementary material for: Toll-Like Receptor 7 Agonist GS-9620 Induces HIV Expression and HIV-Specific Immunity in Cells from HIV-Infected Individuals on Suppressive Antiretroviral Therapy
Source: J Virol. 2017 Mar 29;91(8):e02166-16. doi: 10.1128/JVI.02166-16 (PMC5375698; doi:10.1128/JVI.02166-16)
Supplement: Supplemental material [file supp_91_8_e02166-16__index.html]

Toll-Like Receptor 7 Agonist GS-9620 Induces HIV Expression and HIV-Specific Immunity in Cells from HIV-Infected Individuals on Suppressive Antiretroviral Therapy — Supplemental material 

# Toll-Like Receptor 7 Agonist GS-9620 Induces HIV Expression and HIV-Specific Immunity in Cells from HIV-Infected Individuals on Suppressive Antiretroviral Therapy

## Supplemental material

- Supplemental file 1 -

  Data Set S1 (Individual latency reversal results.)

  XLSX, 74K
